# Effects of Chemical Species and Nonlinear Thermal Radiation with 3D Maxwell Nanofluid Flow with Double Stratification—An Analytical Solution

**DOI:** 10.3390/e22040453

**Published:** 2020-04-16

**Authors:** Iskander Tlili, Sania Naseer, Muhammad Ramzan, Seifedine Kadry, Yunyoung Nam

**Affiliations:** 1 Department for Management of Science and Technology Development, Ton Duc Thang University, Ho Chi Minh City 758307, Vietnam; 2 Faculty of Applied Sciences, Ton Duc Thang University, Ho Chi Minh City 758307, Vietnam; 3 Department of Computer Science, Bahria University, Islamabad 44000, Pakistan; sanianaseer864@gmail.com (S.N.); mramzan@bahria.edu.pk (M.R.); 4 Department of Mechanical Engineering, Sejong University, Seoul 143-747, Korea; 5 Department of Mathematics and Computer Science, Faculty of Science, Beirut Arab University, Beirut 115020, Lebanon; 6 Department of Computer Science and Engineering, Soonchunhyang University, Asan 31538, Korea

**Keywords:** Maxwell nanofluid, chemical reaction, double stratification, nonlinear thermal radiation

## Abstract

This article elucidates the magnetohydrodynamic 3D Maxwell nanofluid flow with heat absorption/generation effects. The impact of the nonlinear thermal radiation with a chemical reaction is also an added feature of the presented model. The phenomenon of flow is supported by thermal and concentration stratified boundary conditions. The boundary layer set of non-linear PDEs (partial differential equation) are converted into ODEs (ordinary differential equation) with high nonlinearity via suitable transformations. The homotopy analysis technique is engaged to regulate the mathematical analysis. The obtained results for concentration, temperature and velocity profiles are analyzed graphically for various admissible parameters. A comparative statement with an already published article in limiting case is also added to corroborate our presented model. An excellent harmony in this regard is obtained. The impact of the Nusselt number for distinct parameters is also explored and discussed. It is found that the impacts of Brownian motion on the concentration and temperature distributions are opposite. It is also comprehended that the thermally stratified parameter decreases the fluid temperature.

## 1. Introduction

Non-Newtonian fluids have extensive applications in industry and engineering sectors. No single relation exists that entirely describes the features of non-Newtonian fluids. Applications depicting the characteristics of non-Newtonian fluids include foams, apple sauce, sugar solution, soaps, clay, and lubricants, etc. Numerous non-Newtonian liquid models are defined by the researchers. Amongst these models, Maxwell liquid is the least complex subclass non-Newtonian liquids model of rate type. This is the broadly used model to investigate the impacts of stress relaxation. Maxwell fluid becomes highly nonlinear and completed in contrast with Newtonian liquid by the inclusion of stress relaxation in the stress tensor when additional stress time is zero. Researchers have shown keen interest to further dig out its novel features. Muhammad et al. [1] debated the flow of boundary layer Maxwell magnetohydrodynamic (MHD) nanofluid in a non-Darcy porous channel, and this flow is produced via a stretched surface. Hussain et al. [2] examined the influence of thermal radiation and double stratification in the flow of Maxwell nano liquid with magnetohydrodynamics. Bai et al. [3] explored MHD stagnation Maxwell nanofluid flow with Brownian motion and thermophoresis effect towards a stretching sheet. Jusoh et al. [4] discussed the three-dimensional flow of MHD Maxwell nanofluids over an extended convectively heated porous extended sheet. Hayat et al. [5] analyzed the nonlinear thermal radiation Maxwell nanofluid flow subjected to a convectively heated stretched surface. The flow of Maxwell fluid with insertion of nanomaterial over an elongated surface which is stretched in an exponential way is analytically studied by Farooq et al. [6]. Series solutions are found locally by bvph 2.0 scheme. They concluded that volumetric concentration in increased for magnetic parameter. Ramzan et al. [7] deliberated numerically upper-convected Maxwell fluid flow past an extended bi-directional stretched sheet under the impacts of nonlinear radiative heat flux and Soret–Dufour effects. It is comprehended from this exploration that concentration of the fluid deteriorates for Soret–Dufour impacts. Some recent studies featuring Maxwell fluid flow may be found at [8,9,10] and many therein.

With the expeditious progress of modern science and technology, nanomaterials are a kind of new material that has gained exclusive attention from numerous researchers. A fluid containing nanometer-sized particles immersed into some base fluid is called nanofluid. The nanoparticles characteristically made of carbon nanotubes, metals or oxides. The excellent features of nanoparticles such as their infinitesimal size and small volume fraction make them highly adequate for the preparation of nanofluids. The nanofluids are different from the ordinary base fluids because they possess extensive enhanced thermal conductivity. These fluids are customarily used in electronics, transportation, fiber-technology, textiles, and energy production. Nanofluid is an ideal applicant to get the title of the working fluid. Rehman et al. [11] explored the thermally magnetized nanofluid flow with heat source/sink. Heat transfer enhancement in free convection flow of carbon nanotubes Maxwell nanofluids with four dissimilar kinds of molecular liquids presented by Aman et al. [12]. The flow of Maxwell nanofluid with viscous dissipation and solar radiation near a stagnation point over an extended surface is studied by Khan et al. [13]. Their findings reveal that role of the thermal radiation effect is dominant in improving the temperature of the fluid. Different investigations have been directed to reveal the various aspects of the nanofluid in [14,15,16,17,18,19,20,21,22,23]. The nanofluids under the influence of magnetohydrodynamics have also numerous interesting and practical applications like optical switches and modulators, drug delivery, optical fiber, and cancer therapy. Numerous explorations in the literature may be seen featuring aspects of these fluids [24,25,26,27,28,29,30,31,32,33].

Stratification has an essential role in heat and mass transfer. Stratification is observed in nanofluids in the form of layers owing to change in concentration or temperature distributions. The simultaneous incidence of heat and mass transfer is the prime justification for double stratification. Many daily life scenarios, such as underground water reservoirs, manufacturing processes, heterogeneous mixtures in the atmosphere, industrial foods, and salinity of oceans encounter stratification. Stratification also plays a significant role in ponds and lakes. In lakes, stratification minimizes by adding oxygen to the base fluid. It maintains a balance in the ratio of oxygen and hydrogen by controlling the temperature and concentration disparities to keep the environment conducive for species evolution. Furthermore, the process of thermal stratification is essential for solar engineering because high energy efficiency can be attained with better stratification. Researchers and scientists are motivated in explorations highlighting the stratification phenomenon. Ibrahim et al. [34] investigated the flow of the viscous nanofluids along with a vertical plate in the existence of double stratifications. Abbasi et al. [35] examined Maxwell fluid flow using mixed convection, the impression of thermal stratification with thermal radiation. Micropolar fluid flow passing a vertical plate with MHD flow of double stratification and mixed convection is discussed by Srinivasacharya et al. [36]. Simultaneous illustration of Jeffrey fluid with thermally radiated and thermally stratified medium via an extended surface is investigated by Hayat et al. [37]. Some more observations about the research thermal and solutal stratification under different perspectives can be found at [38,39,40,41,42,43] many therein.

The effect of non-linear thermal radiation on fluid flows has been conversed by researchers because of its applications, such as in solar ponds, solar collectors, gas turbines, furnace design, photochemical reactors, turbid water bodies, nuclear power plants, and the various propulsion devices for missiles, satellites, aircraft, and space vehicles, as examples of engineering areas. Recently many researchers have explored the thermal radiation impact in heat transfer processes of both conventional and nanofluids over-stretching surfaces. The effects of non-linear thermal radiation and chemical reaction past an Eyring Powell nanofluid flow with variable thermal conductivity are investigated by Ramzan et al. [44]. Radiative magnetohydrodynamic nanofluid flow due to gyrotactic microorganisms with chemical reactions and non-linear thermal radiation is explored by Ramzan et. al [45]. The impact of magnetohydrodynamic on the three-dimensional flow of a viscoelastic nanofluid in the existence of nonlinear thermal radiation is analyzed by Hayat et. al [46]. The Oldroyd-B fluid flow under the influence of radiative heat flux and homogeneous–heterogeneous reactions at a convective heat surface is studied by Lu et al. [47]. It is notified by them that fluid temperature is enhanced versus escalating estimates of Biot number. Bilal et al. [48] analyzed the Hall current impact on a time-dependent rotational fluid flow comprising carbon nanotubes over a convectively heated surface. The additional effects affecting the fluid are nonlinear thermal radiation in a Darry–Forchheimer porous media. Some more recent publications discussing radiative heat flux are referred at [49,50,51,52].

Entropy is the disorder of system and surroundings, for example, spinning movements, kinetic energy, molecular abrasion and friction, transfer of molecules, and others, owing to which wastage of heat occurs and, therefore, a useful heat is unable to transmit completely into work. Because of these additional movements, a disorder in a system and its surroundings are generated. Examples of these processes are mixing of liquids, friction, electrical resistance, chemical reactions, unstained expansion, internal friction, deformation of plastics and unnecessary transfer of heat in a finite temperature difference. Nowadays, entropy analysis is employed as an important tool to find out which procedural system is more efficient and useful for spongy media, electric cooling, turbo engines, and combustions. Quantitative information about the system’s energy can be obtained by the (First) law of thermodynamics. Additionally, the Second law of thermodynamics is found to be a useful mechanism to analyze the entropy generation analysis in order to examine irreversibility related to natural phenomena like that of gas to gas application in a counter flow heat exchanger [53]. With passing time several researchers [54,55,56,57,58,59,60] have discussed entropy with different geometrical configurations.

The homotopy analysis method (HAM) is an analytical approximation method for highly nonlinear equations. The HAM technique is one of the best and simplest techniques for obtaining a convergent series solution for weakly as well as strongly non-linear equations. In 1992, Liao [61] utilized the basic ideas of the homotopy in topology to recommend a general analytical method for nonlinear problems, called the homotopy analysis method (HAM), [62,63,64]. This method has been effectively applied to solve various kinds of nonlinear problems [65,66,67,68,69,70].

In view of the foregoing, it is comprehended that a reasonable number of studies have been conducted in the literature discussing varied aspects of Maxwell nanofluid flow. However, no such study has been conducted so far that deliberates the characteristics of chemical species and heat generation/absorption on a 3D Maxwell nanofluid flow past a bi-directional extended surface. The other attributes adding towards the novelty of the model are magnetohydrodynamics and nonlinear radiative flux with thermal and solutal stratification. A series solution of the envisioned model is attained via the homotopy analysis scheme. The requisite discussions with physical insight are given through graphs.

## 2. Mathematical Modeling

**Figure 1:**
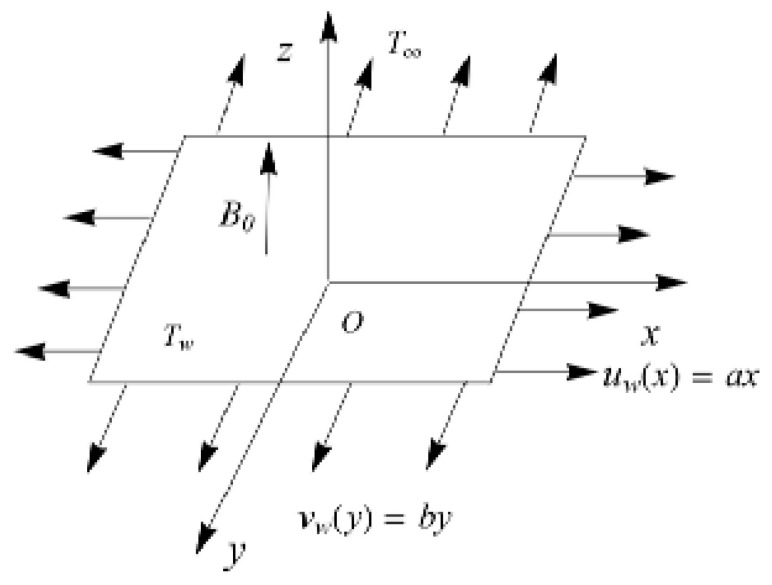
Schematic 3D flow.

Consider a 3D MHD steady Maxwell incompressible nanofluid flow which is non-linearly thermally radiated past a stretching surface. We take a cartesian coordinate system in such a way that the x- and y-axes are directed via the stretching surface while the z-axis is normal to it. Considering $u_{w}\left(x\right)=ax$ and $u_{w}\left(y\right)=bx$ (where a,b are positive real numbers) are the stretching surface velocities towards the directions of x- and y-. Here, the stretching sheet velocities vary linearly at the distance from the origin as assumed in Figure 1.

The resulting boundary layer equations defining Maxwell nanofluid flow with the effects of chemical reaction and non-linear thermal radiation are given below (see reference [71]):

(1) $$u_{x}+v_{y}+w_{z}=0,$$

(2) $$uu_{x}+vu_{y}+wu_{z}+\lambda_{1}\left[\begin{array}{l} u^{2}u_{xx}+v^{2}u_{yy}+w^{2}u_{zz}\\ +2uvu_{xy}+2vwu_{yz}+2uwu_{xz} \end{array}\right]=\nu u_{zz}-\frac{\sigma B_{0}^{2}}{\rho_{f}}(u+\lambda_{1}wu_{z}),$$

(3) $$uv_{x}+vv_{y}+wv_{z}+\lambda_{1}\left[\begin{array}{l} u^{2}v_{xx}+v^{2}v_{yy}+w^{2}v_{zz}\\ +2uvv_{xy}+2vwv_{yz}+2uwv_{xz} \end{array}\right]=\nu v_{zz}-\frac{\sigma B_{0}^{2}}{\rho_{f}}(v+\lambda_{1}wv_{z}),$$

(4) $$uT_{x}+vT_{y}+wT_{z}=\alpha T_{zz}+\frac{Q}{{\left(\rho c\right)}_{f}}(T-T_{\infty })+\tau \left(D_{B}(T_{z}C_{z})+\frac{D_{T}}{T_{\infty }}{\left(T_{z}\right)}^{2}\right)-\frac{1}{\rho c_{p}}{\left(q_{r}\right)}_{z},$$

(5) $$uC_{x}+vC_{y}+wC_{z}=D_{B}\left(C_{zz}\right)+\frac{D_{T}}{T_{\infty }}\left(T_{zz}\right)-k_{r}^{2}\left(C-C_{\infty }\right).$$

with associated boundary conditions:

$$\begin{array}{c} u=ax,\;v=by,\;w=0,\\ T=T_{f}=T_{0}+d_{1}\left(x\right),\;C=C_{f}=C_{0}+d_{2}\left(x\right)\;at\;y=0, \end{array}$$

(6) $$T\to T_{\infty }=T_{0}+e_{1}\left(x\right),\;C\to C_{\infty }=e_{2}\left(x\right)\;\mathrm{at}\;y\to \infty .$$

Via Rosseland approximation, radiative heat flux $q_{r}$ is denoted as [72]:

(7) $$q_{r}=-\frac{4\sigma^{*}}{3k^{*}}\frac{\partial T^{4}}{\partial z}=-\frac{16\sigma^{*}T_{\infty }^{3}}{3k^{*}}\frac{\partial T}{\partial z},$$

writing

(8) $$T^{4}≅4T_{\infty }^{3}T-3T_{\infty }^{4},$$

Invoking Equation (7) in Equation (4), the temperature takes the form:

(9) $$uT_{x}+vT_{y}+wT_{z}={\left[\left(\alpha +\frac{1}{\rho c_{p}}\frac{16\sigma^{*}T_{\infty }^{3}}{3k^{*}}\right)T_{z}\right]}_{z}+\frac{Q}{{\left(\rho c\right)}_{f}}\left(T-T_{\infty }\right)+\tau \left[D_{B}(T_{z}C_{z})+\frac{D_{T}}{T_{\infty }}{\left(T_{z}\right)}^{2}\right].$$

The dimensionless form of the envisioned mathematical model is found by applying the following transformations [73]:

(10) $$\begin{array}{l} u=axf^{\prime }\left(\eta \right),\;v=ayg^{\prime }\left(\eta \right),\;w=-{\left(a\nu \right)}^{1/2}\left(f\left(\eta \right)+g\left(\eta \right)\right),\\ \;\eta ={\left(\frac{a}{\nu }\right)}^{1/2}z,\;\theta \left(\eta \right)=\frac{T-T_{\infty }}{T_{f}-T_{0}},\;\phi \left(\eta \right)=\frac{C-C_{\infty }}{C_{\infty }}. \end{array}$$

Which satisfies the continuity Equation (1) while Equations (2) to (6) are as follows:

(11) $$f^{\prime \prime \prime }+(M^{2}\beta_{1}+1)(f+g)f^{\prime \prime }-f^{\prime }{}^{2}+\beta_{1}\left(2(f+g)f^{\prime }f^{\prime \prime }-{\left(f+g\right)}^{2}f^{\prime \prime \prime }\right)-M^{2}f^{\prime }=0,$$

(12) $$g^{\prime \prime \prime }+(M^{2}\beta_{1}+1)(f+g)g^{\prime \prime }-g^{\prime }{}^{2}+\beta_{1}\left(2(f+g)g^{\prime }g^{\prime \prime }-{\left(f+g\right)}^{2}g^{\prime \prime \prime }\right)-M^{2}g^{\prime }=0,$$

(13) $$\begin{array}{l} \theta^{\prime \prime }+(f+g)\mathrm{Pr}\theta^{\prime }-\mathrm{Pr}\left(S_{1}+\theta \right)f^{\prime }+\frac{Nb}{Nt}\mathrm{Pr}\theta^{\prime }\phi^{\prime }+\mathrm{Pr}\theta^{\prime }{}^{2}+\mathrm{Pr}S\theta \\ +\frac{4}{3}Rd\left[\begin{array}{l} \left({\left(\theta_{w}-1\right)}^{3}\theta^{3}\theta^{\prime \prime }\right)+3{\left(\theta_{w}-1\right)}^{2}\theta^{2}\theta^{\prime \prime }+\theta^{\prime \prime }+3{\left(\theta_{w}-1\right)}^{3}\theta^{\prime }{}^{2}\theta^{2}\\ +3(\theta_{w}-1)\theta^{\prime \prime }\theta +3(\theta_{w}-1)\theta^{\prime }{}^{2}+6{\left(\theta_{w}-1\right)}^{2}\theta^{\prime }{}^{2}\theta \end{array}\right]=0, \end{array}$$

(14) $$\phi^{\prime \prime }+\frac{Nt}{Nb}\theta^{\prime \prime }+Sc(f+g)\phi^{\prime }-f^{\prime }\left(S_{2}+\phi \right)Sc-k\phi =0.$$

With

$$f\left(0\right)=g\left(0\right)=0,\;f^{\prime }\left(0\right)=1,\;g^{\prime }\left(0\right)=\lambda ,\;\theta^{\prime }\left(0\right)=\left(1-S_{1}\right),\;\phi^{\prime }\left(0\right)=\left(1-S_{2}\right),$$

(15) $$f^{\prime }\left(\infty \right)\to 0,\;g^{\prime }\left(\infty \right)\to 0,\;\theta \left(\infty \right)\to 0,\;\phi \left(\infty \right)\to 0.$$

The distinct dimensionless parameters arising in the above equations are characterized as:

(16) $$\begin{array}{l} \beta_{1}=\lambda_{1}a,M^{2}=\frac{\sigma B_{0}^{2}}{\rho_{f}a},Nb=\frac{\tau D_{B}C_{\infty }}{\nu },Nt=\frac{\tau D_{T}(T_{f}-T_{0})}{\nu T_{\infty }},S=\frac{Q}{a{\left(\rho c\right)}_{f}},\\ Rd=\frac{4\sigma^{*}T_{\infty }^{3}}{Kk^{*}},Sc=\frac{\nu }{D_{B}},\mathrm{Pr}=\frac{\nu }{\alpha },k=\frac{k_{r}^{2}}{a},S_{1}=\frac{e_{1}}{d_{1}},S_{1}=\frac{e_{1}}{d_{1}}. \end{array}$$

The dimensional form of heat transfer rate and Skin friction coefficients are appended below:

(17) $$C_{fx}=\frac{\tau_{wx}}{\rho u_{w}^{2}\left(x\right)},\;C_{fy}=\frac{\tau_{wy}}{\rho u_{w}^{2}\left(y\right)},\;Nu_{x}=\frac{xq_{w}}{K\left(T_{f}-T_{\infty }\right)},$$

where

(18) $$\begin{array}{c} \tau_{wx}=\mu \left(1+\beta_{1}\right){\left(u_{z}\right)}_{z=0},\;\tau_{wy}=\mu \left(1+\beta_{1}\right){\left(v_{z}\right)}_{z=0},\\ q_{w}=-K{\left(T_{z}\right)}_{z=0}+{\left(q_{r}\right)}_{w}. \end{array}$$

The local Nusselt number and skin friction coefficients in dimensionless form are as under:

(19) CfxRex1/2=(1+β1)f″(0),NuxRex−12=−[1+Rd{1+(θw−1)θ(0)}3]θ′(0),

where the local Reynolds number is represented by Rex=uwxν.

## 3. Solution by HAM

The acceptable initial approximations and corresponding linear operators are constructed as:

(20) $$f_{0}\left(\eta \right)=1-e^{-\eta },\;g_{0}\left(\eta \right)=\lambda \left(1-e^{-\eta }\right),\;\theta_{0}\left(\eta \right)=\left(1-S_{1}\right),\;\phi_{0}\left(\eta \right)=\left(1-S_{2}\right),\;L_{f}=f^{\prime \prime \prime }-f^{\prime },\;L_{g}=g^{\prime \prime \prime }-g^{\prime },\;L_{\theta }=\theta^{\prime \prime }-\theta ,\;L_{\phi }=\phi^{\prime \prime }-\phi .$$

The linear operators mentioned above obey the below-mentioned properties:

(21) $$\begin{array}{l} L_{f}\left\{B_{1}+B_{2}e^{\eta }+B_{3}e^{-\eta }\right\}=0,\;L_{g}\left\{B_{4}+B_{5}e^{\eta }+B_{6}e^{-\eta }\right\}=0,\\ L_{\theta }\left\{B_{7}e^{\eta }+B_{8}e^{-\eta }\right\}=0,\;L_{\phi }\left\{B_{9}e^{\eta }+B_{10}e^{-\eta }\right\}=0. \end{array}$$

where the arbitrary constants are represented by $B_{j}\left(j=1\;\mathrm{to}\;10\right).$

## 4. Entropy Generation Analysis

Maxwell fluid volumetric entropy equation is represented by:

(22) $$\begin{array}{l} S_{gen}^{\prime \prime \prime }=\underset{HTI}{\underset{︸}{\frac{k}{T_{\infty }^{2}}\left[1+\frac{16\sigma^{*}T_{\infty }^{3}}{3Kk^{*}}\right]{\left(T_{z}\right)}^{2}}}+\underset{FFI}{\underset{︸}{\frac{\mu }{T_{\infty }}\left[{\left(u_{z}\right)}^{2}+{\left(v_{z}\right)}^{2}\right]+\frac{\sigma B_{0}^{2}}{T_{\infty }}\left[u^{2}+v^{2}\right]}}\\ +\underset{DI}{\underset{︸}{\frac{RD}{C_{\infty }}{\left(C_{z}\right)}^{2}+\frac{RD}{T_{\infty }}\left(T_{z}C_{z}\right)}}. \end{array}$$

Entropy generation comprises of three impacts which are present in above equation i.e.,

- Fluid friction irreversibility (FFI)
- Diffusive irreversibility (DI)
- Conduction effect or heat transfer irreversibility (HTI)

The characteristic of entropy generation is formulated as:

(23) $$S_{0}^{\prime \prime \prime }=\frac{{\left(\Delta T\right)}^{2}K}{L^{2}T_{\infty }^{2}}.$$

(24) $$\mathrm{The}\;\mathrm{entropy}\;\mathrm{generation}\;\mathrm{number}\;N_{s}\mathrm{is}\;\mathrm{characterized}\;\mathrm{as}:\;N_{s}=\frac{S_{gen}^{\prime \prime \prime }}{S_{o}^{\prime \prime \prime }}$$

The dimensionless form of entropy number is given by:

(25) $$\begin{array}{l} N_{S}=\frac{S_{gen}^{\prime \prime \prime }}{S_{0}^{\prime \prime \prime }}=Re\left(1+\frac{4}{3}Rd\right)\theta^{\prime }{}^{2}+ReBr\left[{\left(f^{\prime \prime }\right)}^{2}+\xi^{2}{\left(g^{\prime \prime }\right)}^{2}\right]+\frac{BrHa^{2}}{\Omega }\left[{\left(f^{\prime }\right)}^{2}+\xi^{2}{\left(g^{\prime }\right)}^{2}\right]\\ +\frac{Re}{\Omega^{2}}\sum {\left(\phi^{\prime }\right)}^{2}+\frac{Re\sum }{\Omega }\theta^{\prime }\phi^{\prime }. \end{array}$$

The parameters defined in Equation (25) are translated in the following form:

(26) $$Re=\frac{aL^{2}}{\nu },\;Br=\frac{\mu u_{w}{}^{2}}{K\Delta T},\;\Omega =\frac{\Delta T}{T_{\infty }},\;Ha=B_{0}^{2}L^{2}\sqrt{\frac{\sigma }{\mu }}\;,\sum =\frac{RDC_{\infty }}{K},\;\xi =\frac{y}{x}.$$

## 5. Convergence Analysis

The homotopy analysis technique is applied to acquire series solutions, and these solutions depend highly on supplementary parameters $\hbar_{f},\;\hbar_{g},\;\hbar_{\theta }\;and\;\hbar_{\phi }$. The series solutions of convergence region can be regulated with the assistance of these parameters. The ranges of these parameters are $-1.6\leq \hbar_{f}\leq -0.2,\;-1.5\leq \hbar_{g}\leq -0.3,\;-1.7\leq \hbar_{\theta }\leq -0.8,\;and-1.6\leq \hbar_{\phi }\leq -0.1.$

**Figure 2:**
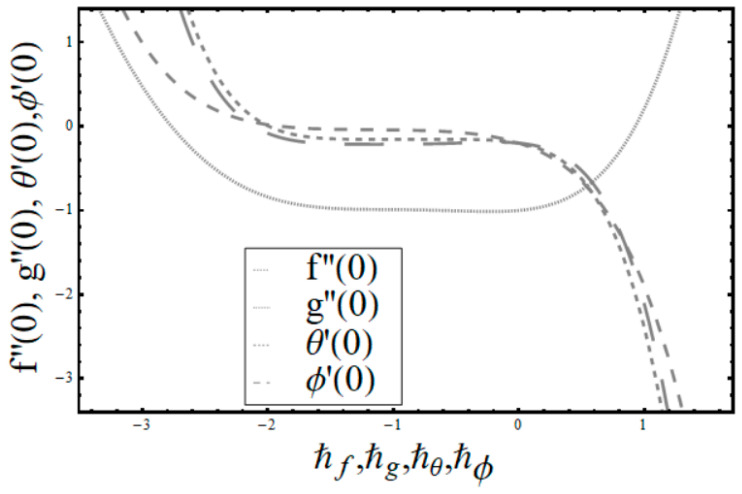
ℏ- curves for f,g,θ,ϕ.

Table 1 is established to see that $25^{\mathrm{th}}$ approximation is appropriate for three distributions. The values obtained in Table 1 are in total alignment to the curves shown in Figure 2. A magnificent correlation between the graphical and numerical outcomes is seen.

## 6. Results with Discussion

**Figure 3:**
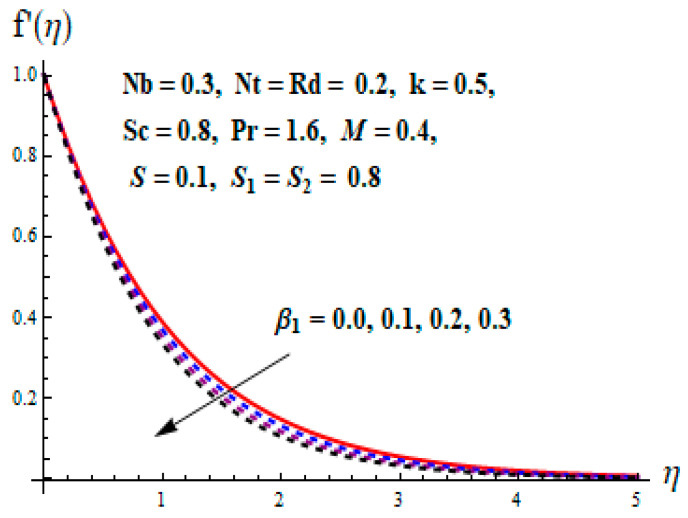
Change of $\beta_{1}$ versus $f^{\prime }$.

**Figure 4:**
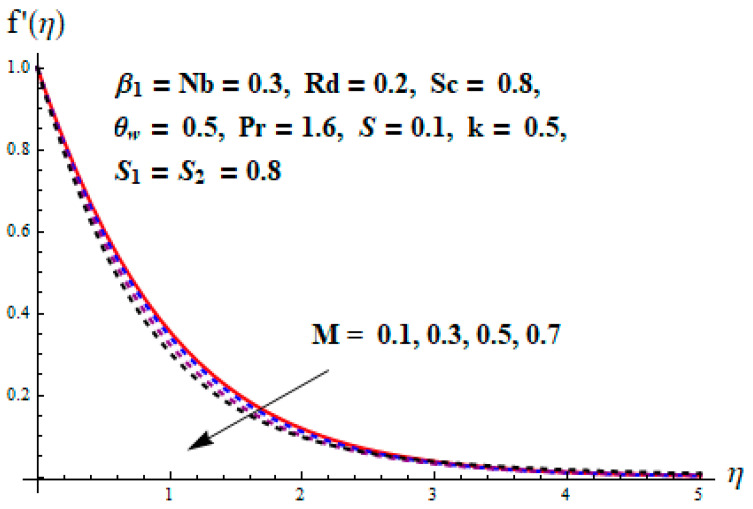
Change of M versus $f^{\prime }$.

**Figure 5:**
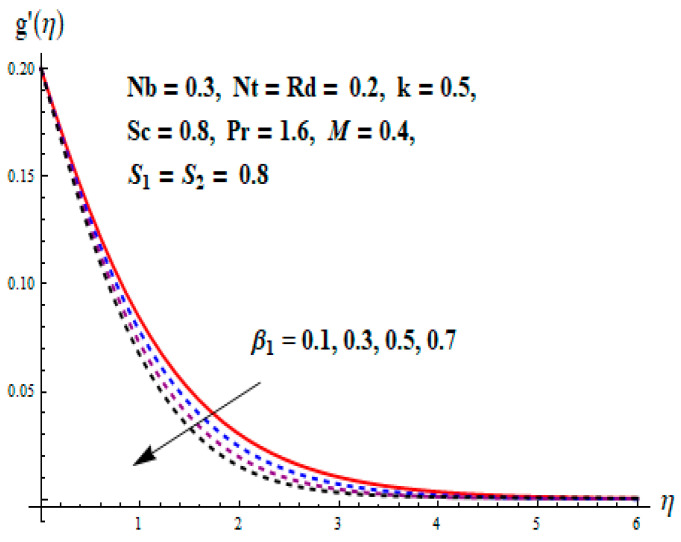
Change of $\beta_{1}$ versus $g^{\prime }$.

**Figure 6:**
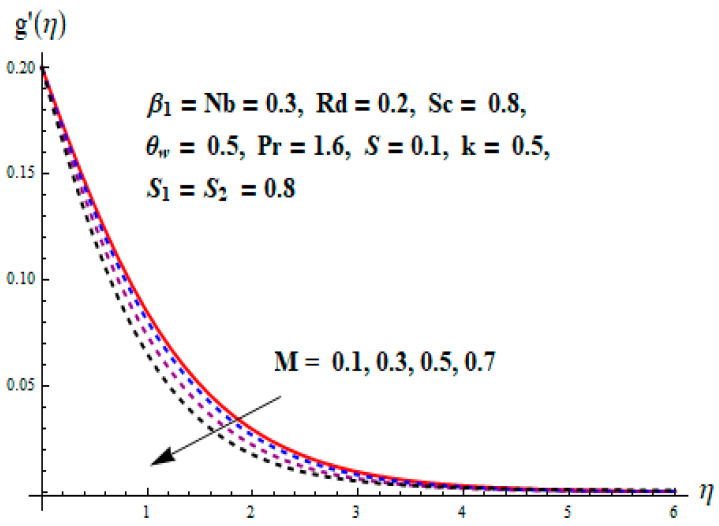
Change of M versus $g^{\prime }$.

**Figure 7:**
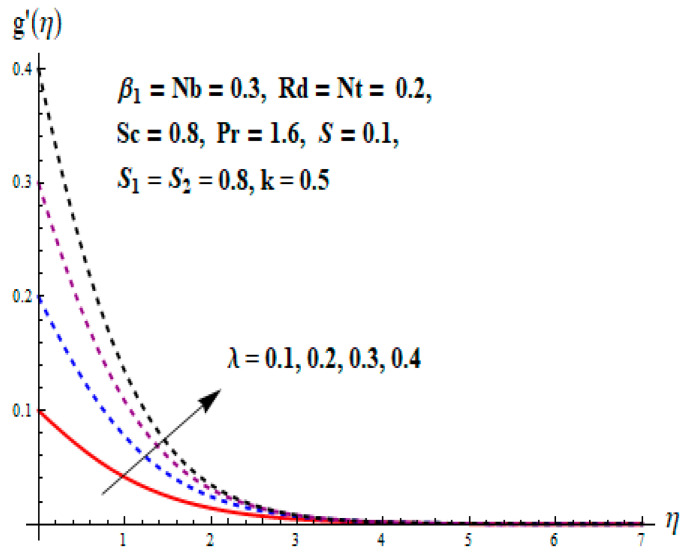
Change of λ versus $g^{\prime }$.

**Figure 8:**
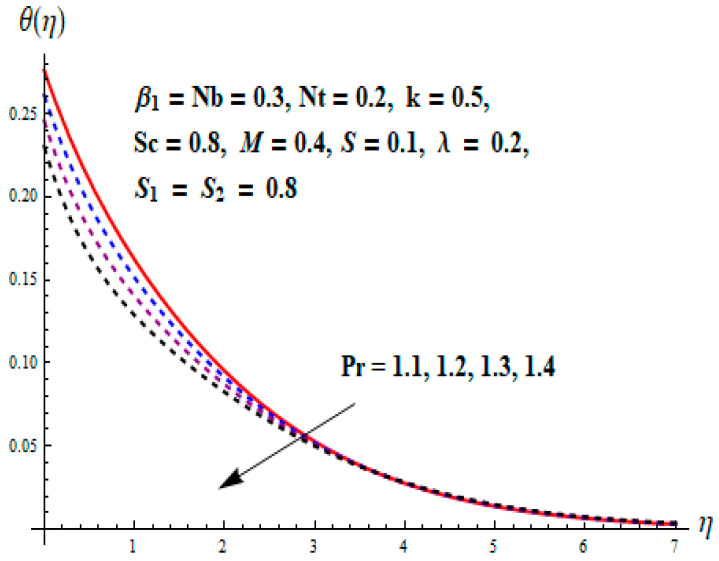
Change of Pr versus θ.

**Figure 9:**
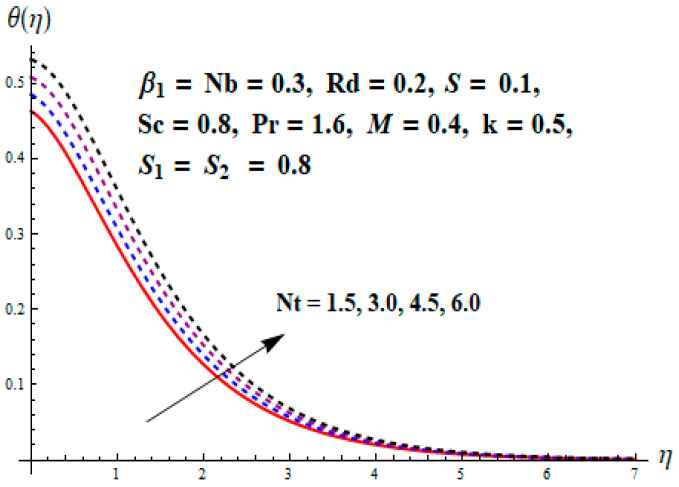
Change of Nt versus θ.

**Figure 10:**
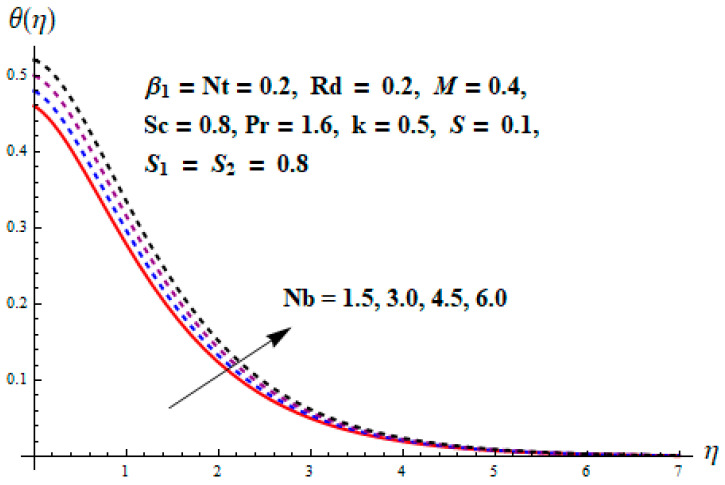
Change of Nb versus θ.

**Figure 11:**
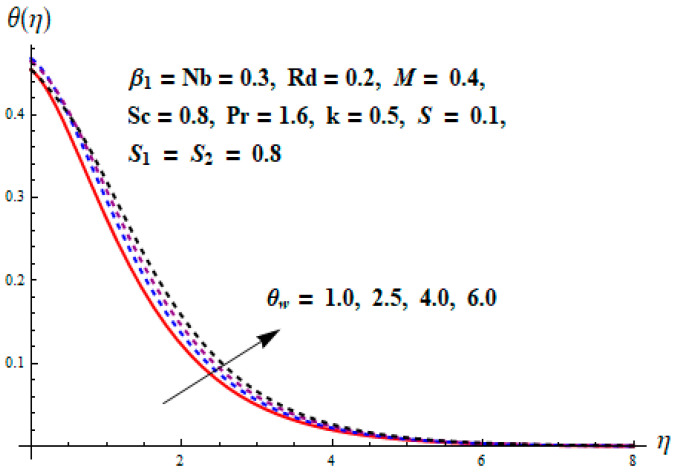
Change of $\theta_{w}$ versus θ.

**Figure 12:**
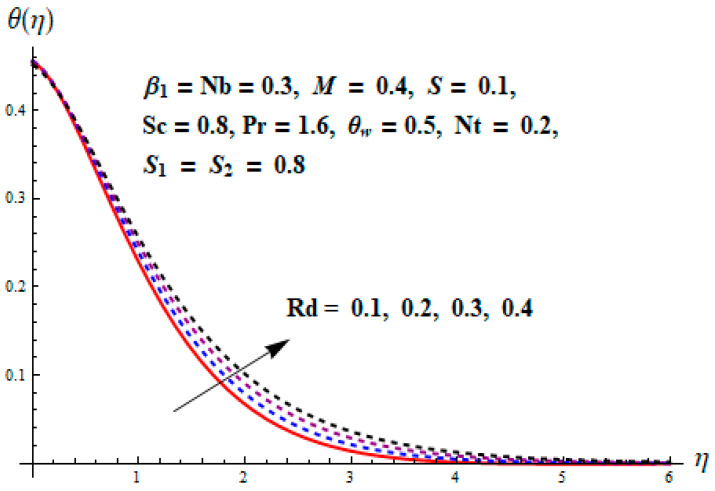
Change of Rd versus θ.

**Figure 13:**
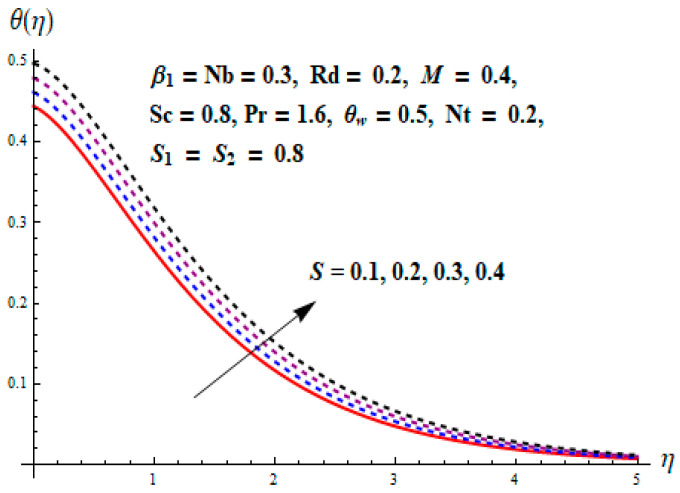
Change of S versus θ.

**Figure 14:**
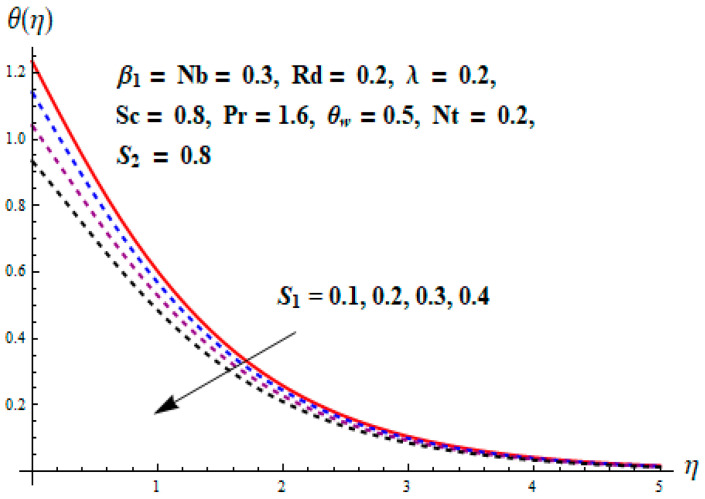
Change of $S_{1}$ versus θ.

**Figure 15:**
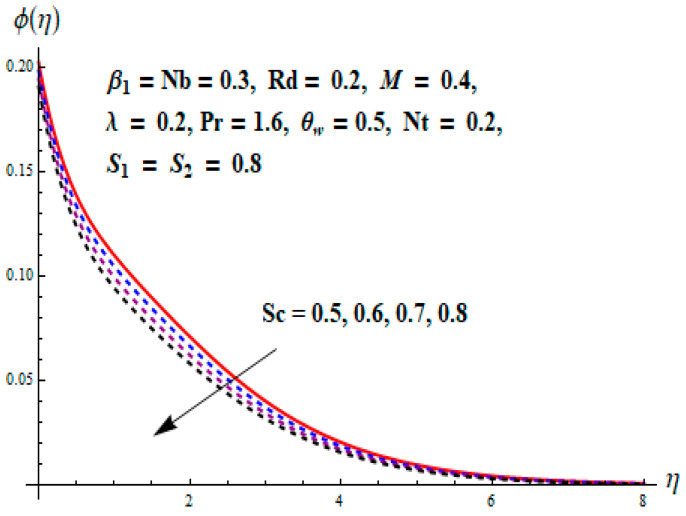
Change of Sc versus ϕ.

**Figure 16:**
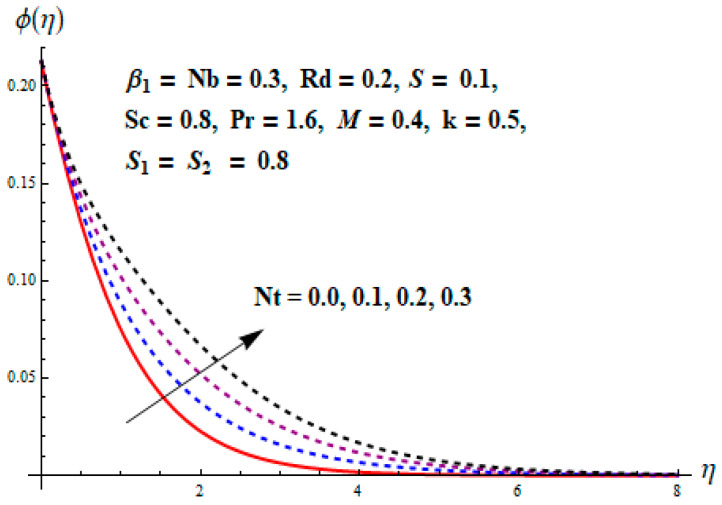
Change of Nt versus ϕ.

**Figure 17:**
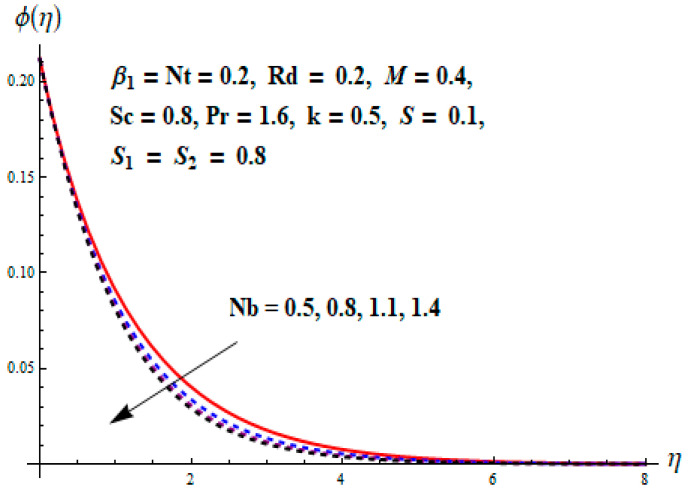
Change of Nb versus ϕ.

**Figure 18:**
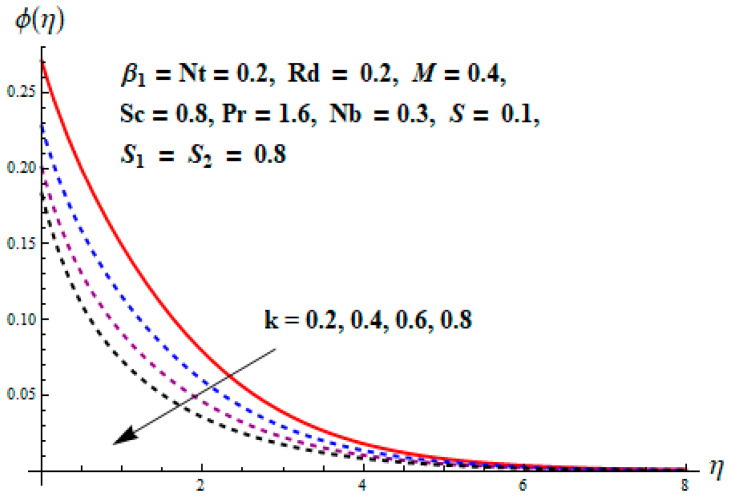
Change of k versus ϕ.

**Figure 19:**
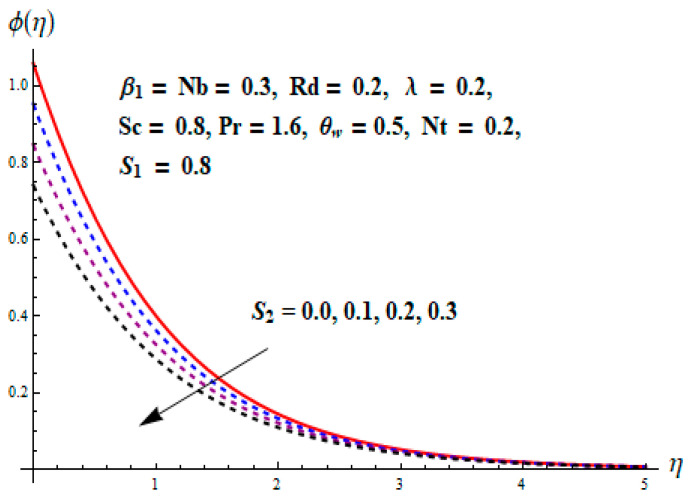
Change of $S_{2}$ versus ϕ.

**Figure 20:**
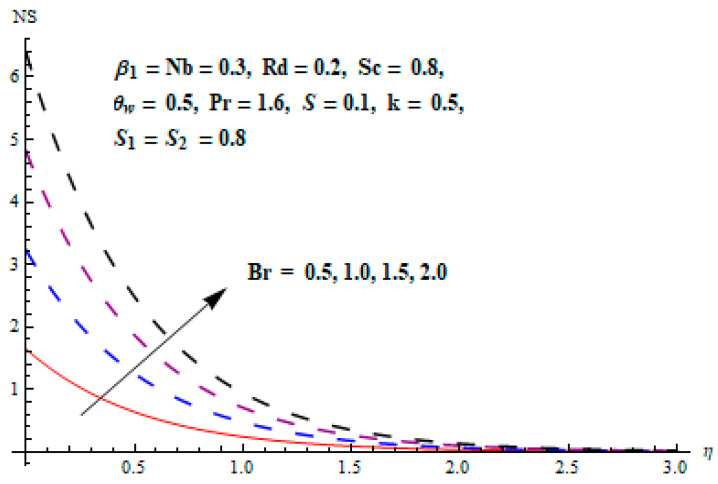
Change of Br versus Ns.

**Figure 21:**
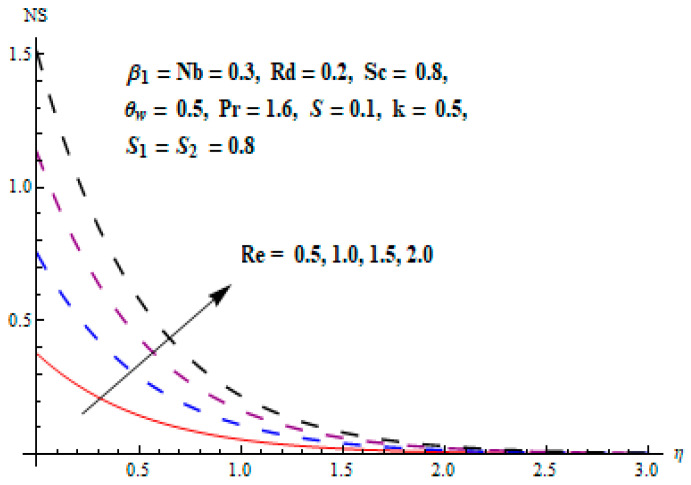
Change of Re versus Ns.

This portion focuses to discuss the important attributes of parameters versus velocity, concentration and temperature distributions. Various parameters like the magnetic parameter M, Schmidt number Sc, Deborah number $\beta_{1}$, Prandtl number Pr, Brownian motion parameter Nb, parameter of heat absorption/generation S, parameter of thermophoresis Nt, parameter of temperature ratio $\theta_{w}$, chemical reaction parameter k and parameter of radiation Rd are discussed versus velocity, temperature and concentration profiles. Deborah number $\beta_{1}$ effect on the velocity distribution is drawn in Figure 3. With an increment in $\beta_{1}$, velocity profile $f^{\prime }\left(\eta \right)$ decreases. When Deborah number enhances, consequently more resistance to the flow of the fluid is observed that eventually lowers the fluid velocity. Features of magnetic parameter M on $f^{\prime }\left(\eta \right)$ are sketched in Figure 4. We concluded that the $f^{\prime }\left(\eta \right)$ decreases for large M. Relatively larger estimates of M results in strong Lorentz force that obstructs the movement of the fluid flow. Thus, the decline in the velocity of the fluid is witnessed. Effects of Deborah number $\beta_{1}$ on velocity distribution along vertical axis i.e., $g^{\prime }\left(\eta \right)$ are shown in Figure 5. Velocity decreases for larger $\beta_{1}$. As, $\beta_{1}$ is the proportion of relaxation and observation times, so relaxation time increase when Deborah number enhances. Consequently, more resistance provided to the flow of the fluid that lowers the velocity. The case $\beta_{1}=0,$ reflects Newtonian fluid. Figure 6 is drawn to show the impact of M on the velocity profile $g^{\prime }\left(\eta \right)$ along the vertical axis. Here, the same logic may be added as in the case of Figure 4. Further, M=0 leads to a hydrodynamic flow situation. Change in the velocity field $g^{\prime }\left(\eta \right)$ for various estimations of ratio parameter λ is drawn in Figure 7. For mounting estimations of ratio parameter λ, thickness and velocity of the thermal layer shows the higher behavior. Figure 8 demonstrates the effect of Prandtl number Pr on temperature profile θ(η). We found that the profile becomes lower for large variations of Pr. As Pr represents the rate of thermal and momentum diffusivities. Increment in Prandtl number enhances the momentum diffusivity and declines the thermal diffusivity. That affects the temperature of the fluid and eventually decay in the temperature of the fluid is witnessed. Figure 9 is drawn to portray the impact of the thermophoresis parameter Nt on temperature profile θ(η). When the value of Nt is enhanced, the temperature and thermal layer thickness both increases. If we increase the thermophoresis parameter Nt, it causes a raise in the force of thermophoresis, which pushes the nanoparticles from hotter to the colder surface, thus an improvement in the temperature of the fluid is seen. Figure 10 illustrates an upsurge in the temperature profile versus increment in the Brownian motion parameter Nb. The motion of fluid particles rises when Nb enhances. As a result, more heat generates which boosts the temperature. Figure 11 depicts the behavior of fluid temperature for the temperature ratio parameter $\theta_{w}$. Here θ(η) is increasing $\theta_{w}$. Figure 12 plotted to show the curves of θ(η) for various estimates of radiation parameter Rd. Generally, in the process of radiation, heat is generated in the moving liquid, which causes a rise in the thermal field. Figure 13 indicates distinct estimations of heat absorption/generation parameter S for different variations in temperature profile θ(η). Here, S>0 leads to the generation of heat and S<0 correspond to the absorption of heat. It is seen that values of θ(η) and thickness of the thermal layer is higher in the case of heat generation as we see in the situation of heat absorption. Figure 14 is drawn to examine the temperature difference for various variations of thermal stratification parameter $S_{1}$. Here, we noticed that the temperature is a diminishing function of $S_{1}$. Figure 15 is plotted to show that by increasing Schmidt number Sc decay in concentration distribution found. The coefficient of Brownian diffusion and the Schmidt number is related inversely with each other. Greater estimations of Schmidt number Sc give rise to a lower coefficient of Brownian diffusion, which reduces the concentration distribution of nanoparticles. Figure 16 displays the increasing impact of thermophoresis parameter Nt on the concentration profile ϕ(η). The thermophoresis is inversely related to the viscous force. Stronger Nt means the weaker viscous forces. Thus, a rise in the concentration of the fluid is seen. The descending behavior in ϕ(η) for a parameter of Brownian motion Nb is drawn in Figure 17. The Brownian motion arises due to the flow of a nanofluid in the existence of nanoparticles. An increment in Nb affects the Brownian motion, and it reduces the boundary layer thickness. Figure 18 describes the behavior of concentration field versus chemical reaction parameter k. Mounting values of chemical reaction parameter k relate to the reduction in nanoparticle distribution. Figure 19 indicates the significant aspects of solutal or concentration stratification parameter $S_{2}$ on ϕ(η). Concentration relates the decaying nature with the intensity of solutal stratification. Because of a large disturbance in molecules of fluid, the reduction in solute nanoparticle concentration occurs which causes decay in concentration profile ϕ(η). Figure 20 and Figure 21 show important characteristics of volumetric entropy generation versus Brinkman number Br and Reynold number Re respectively. The Brinkman number Br predicts the relative significance of resistive impacts and as it is witnessed that the increment in Br results in entropy increase. The Reynolds number also plays a crucial part in generating higher entropy. The enhancement of Reynolds number backs the contribution of the entropy generation number due to liquid friction and the transfer of heat in the boundary layer. Therefore, the increase in Reynolds number interrupts the liquid flow, and then chaos (disturbance) appears in the liquid.

**Figure 22:**
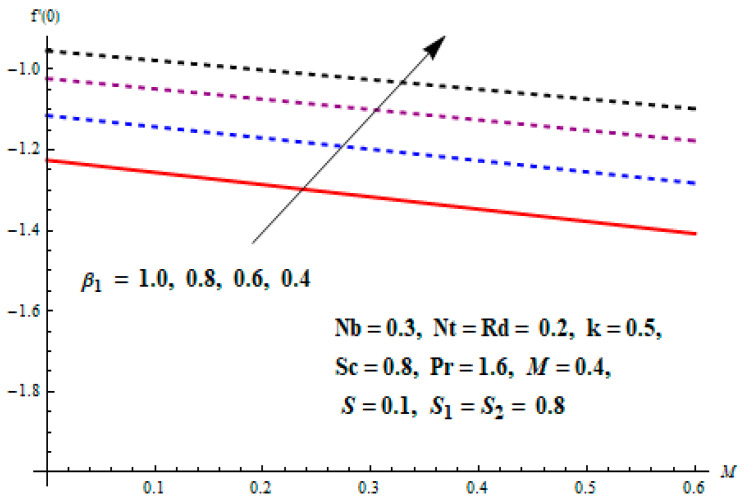
Change of M and $\beta_{1}$ on skin friction.

**Figure 23:**
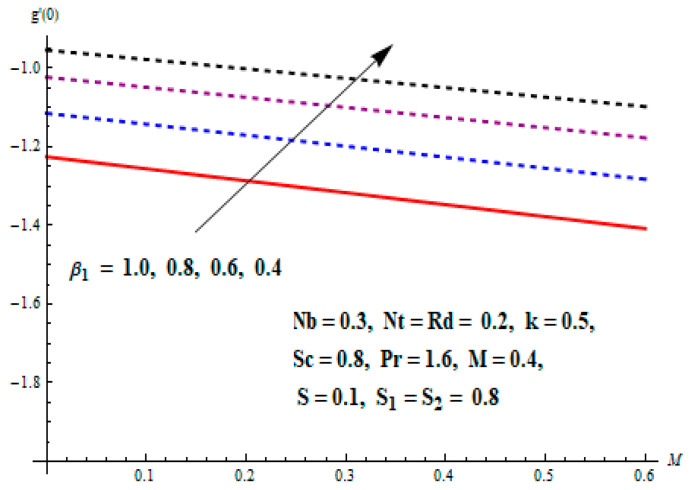
Change of M and $\beta_{1}$ on skin friction.

**Figure 24:**
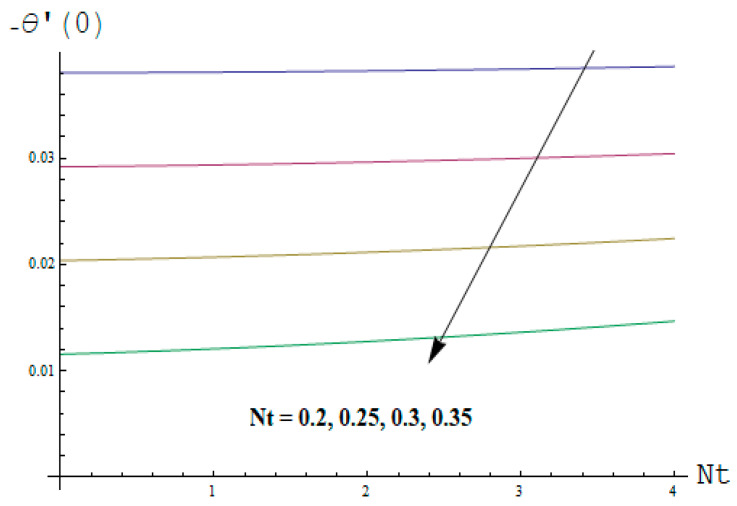
Change of NuxRex−12 versus Nt and Pr.

Effects of Deborah number with respect to relaxation time $\beta_{1}$ and magnetic parameter M on Skin friction coefficient of velocity distributions are portrayed in Figure 22 and Figure 23. It is perceived that Skin friction coefficient is an increasing function of $\beta_{1}$ and M in both cases. The influence of Nt and Pr on heat transfer rate is shown in Figure 24. It can be seen that the heat transfer rate decreases with the increasing values of Nt and Pr.

Table 2 represents the comparison with the benchmark solution Bilal et al. [71] in limiting case for numerous estimates of selected parameters. An excellent harmony in the two solutions is achieved.

## 7. Concluding Remarks

In the current exploration, we have studied the 3D, MHD Maxwell flow of nanofluid with the effects of chemical reaction, higher-order thermal radiation, and double stratification. A homotopy technique is applied for the analytical results. The major findings of the current investigation are appended as follows:

➢ Impacts of Brownian motion on the concentration and temperature distributions are different.
➢ Strong magnetic impact hinders the fluid velocity.
➢ Enhancing the estimation of Deborah’s number will lead to an enhancement in the velocity distributions.
➢ Concentration profile declines for the large estimates of the chemical reaction parameter.
➢ The thermally stratified parameter reduces the temperature field.
➢ Fluid temperature is enhanced for nonlinear radiative heat flux.
➢ The parameter of solutal stratification decreases the concentration distribution.
➢ The heat transfer rate reduces for mounting estimations of Nt and Pr.
➢ Large estimates of Reynolds and Brinkman numbers boost the entropy of the system.

## Figures and Tables

**Table 1:** The analytical solution’s convergence for varying order of approximations while $S=0.1,\;\beta_{1}=0.3$, Nt=0.1, Pr=1.0, Nb=0.3, Rd=0.2, Sc=0.8, M=0.4, $\theta_{w}=0.1$, λ=0.2, $S_{1}=0.8$ and $S_{2}=0.8$.

Approximations Order	$-f^{\prime \prime }\left(0\right)$	$-g^{\prime \prime }\left(0\right)$	$-\theta^{\prime }\left(0\right)$	$-\phi^{\prime }\left(0\right)$
1	0.99750	0.15980	0.13620	0.18150
5	1.00200	0.15390	0.09212	0.20190
10	0.99880	0.15360	0.07378	0.21070
20	0.99660	0.15400	0.06439	0.21430
25	0.99660	0.15400	0.06439	0.21430

**Table 2:** Comparison with Bilal et al. [71] for the values of temperature gradient versus varied estimates of $\beta_{1}$, λ, Pr, $\theta_{w}$, and Rd in limiting case.

$\beta_{1}$	λ	Pr	Rd=0.0				Rd=1.0			
			$\theta_{w}=0$		$\theta_{w}=0$		$\theta_{w}=1.1$		$\theta_{w}=1.5$	
			Reference [71]	Present	Reference [71]	Present	Reference [71]	Present	Reference [71]	Present
1	0.5	2	1.0169493	1.0169463	0.6127148	0.6127137	0.5395022	0.5395012	0.3170918	0.3170911
		4	1.6016506	1.6016498	1.0169493	1.0169473	0.9034844	0.9034812	0.5510692	0.5510653
		7	2.2439298	2.2439277	1.4727082	1.4727065	1.3143535	0.3143525	0.8208336	0.8208313
		10	2.7550740	2.7550733	1.8369216	1.8369208	1.6428361	1.6428345	1.0373929	1.0373912
1	0	7	1.8260260	1.8260243	1.2025347	1.2025333	1.0737145	1.0737134	0.6725491	0.6725478
	0.3		2.0940290	2.0940256	1.3782398	1.3782376	1.2305095	1.2305067	0.7701323	0.7701314
	0.6		2.3129240	2.3129212	1.5154397	1.5154387	1.3609931	1.3609911	0.8434082	0.8434046
	1		2.5591800	2.5591777	1.6644187	1.6644165	1.4836290	1.4836267	0.9204030	0.9204012
0	0.5	7	2.3543502	2.3543499	1.5922506	1.5302056	1.4275931	1.4275911	0.9108748	0.9108735
0.5			2.2966491	2.2966478	1.5302076	1.5302054	1.3684014	1.3684004	0.8638003	0.8638000
1.0			2.2439499	2.2439477	1.4727082	1.4727057	1.3143533	1.3143501	0.8208334	0.8208325
1.5			2.1950047	2.1950034	1.4200796	1.4200773	1.2649215	1.2649201	0.7818260	0.7818245

## Nomenclature

x,y,z	Coordinate axis
u,v,w	Velocity components
$B_{0}$	Magnetic field strength
μ	Dynamic viscosity
$\lambda_{1}$	Fluid parameter
$\rho_{f}$	Base fluid’s density
$q_{r}$	Radiative heat flux
ν	Kinematic viscosity
${\left(\rho c\right)}_{f}$	Fluid’s heat capacity
Q	Heat generation/absorption coefficient
T	Fluid temperature
$c_{p}$	Specific heat capacity
α	Thermal diffusivity of fluid
K	Thermal conductivity
$T_{\infty }$	Temperature away from the surface
C	Nanoparticles concentration
$C_{\infty }$	Concentration away from the surface
$k^{*}$	Coefficient of mean absorption
$Re_{x}$	Local Reynolds number
$k_{r}$	Reaction rate
$C_{0}$	Reference concentration
$T_{f}$	Convective fluid temperature
$C_{f}$	Convective fluid concentration
$\sigma^{*}$	Stefan-Boltzman constant
$q_{w}$	Wall heat flux
$D_{T}$	Thermophoretic diffusion coefficient
k	Chemical reaction parameter
M	Magnetic parameter
$\beta_{1}$	Deborah number expressing relaxation time
$\theta_{w}$	Ratio parameter for temperature
${\left(\rho c\right)}_{p}$	Effective heat capacity of nanoparticles
$D_{B}$	Brownian diffusivity coefficient
S	Heat absorption/generation parameter
θ	Non-dimensional temperature
ϕ	Non-dimensional concentration
f	Non-dimensional velocity
Sc	Schmidt number
η	Similarity variable
$T_{0}$	Reference temperature
Nb	Brownian motion
Nt	Thermophoresis parameter
Pr	Prandtl number
Rd	Parameter for radiation
$Nu_{x}$	Nusselt number
$S_{1}$	Parameter of thermal stratification
$S_{2}$	Parameter of concentration stratification
Br	Brinkmann number
$N_{s}$	Dimensionless entropy number
$S_{0}^{\prime \prime }$	Dimensionless entropy generation rate
Ha	Hartmann number
$S_{gen}^{\prime \prime }$	Volumetric entropy generation per unit length
